# Antioxidant Effects of *Apocynum venetum* Tea Extracts on d-Galactose-Induced Aging Model in Mice

**DOI:** 10.3390/antiox8090381

**Published:** 2019-09-08

**Authors:** Chong Li, Fang Tan, Jianjun Yang, Yue Yang, Yuting Gou, Shuting Li, Xin Zhao

**Affiliations:** 1 Chongqing Collaborative Innovation Center for Functional Food, Chongqing University of Education, Chongqing 400067, China (C.L.) (J.Y.) (Y.Y.) (Y.G.) (S.L.); 2 Chongqing Engineering Research Center of Functional Food, Chongqing University of Education, Chongqing 400067, China; 3 Chongqing Engineering Laboratory for Research and Development of Functional Food, Chongqing University of Education, Chongqing 400067, China; 4 Department of Public Health, Our Lady of Fatima University, Valenzuela 838, Philippines; 5 College of Biological and Chemical Engineering, Chongqing University of Education, Chongqing 400067, China

**Keywords:** *Apocynum venetum* tea extracts, d-galactose, mice, oxidative damage, biological components

## Abstract

As a traditional Chinese medicinal drink, *Apocynum venetum*, a local tea from Xinjiang, China, is favored for its rich flavor and biological functionality. This study looked at aging mice induced by d-galactose to determine the in vivo anti-aging effect of *Apocynum venetum* tea extracts (AVTEs) and its bioactive components. We evaluated the weight of major organs (via organ index) and pathological changes in the liver. We also detailed the effects of AVTE (250 mg/kg in the low dose group, 500 mg/kg in the high dose group) on biochemical parameters (malondialdehyde, superoxide dismutase, glutathione, glutathione peroxidase, catalase, total antioxidant capacity, and nitric oxide) and cytokines (IL-6, IL-12, TNF-α and IL-1β) in the serum of aging mice. We investigated the anti-aging effects of AVTE in d-galactose-induced aging mice via quantitative real-time reverse transcription-polymerase chain reaction (RT-qPCR) assay. In addition, we analyzed the biological components of AVTEs by high performance liquid chromatography (HPLC). The results were remarkable, suggesting that AVTE significantly improved d-galactose-induced aging mice, with the high dose group showing the best results among other groups. ATVE can effectively alleviate hepatocyte edema, as well as inflammatory cell infiltration and injury in mice, induce a protective effect via up-regulation of glutathione (GSH), glutathione peroxidase (GSH-Px), superoxide dismutase (SOD), and catalase (CAT) antioxidant related factors, and play an important role in the up-regulation of anti-inflammatory factors (IL-10) and the down-regulation of pro-inflammatory factors (IL-6, TNF-α and IL-1β). At the same time, HPLC analysis showed that AVTEs contain neochlorogenic acid, chlorogenic acid, cryptochlorogenic acid, rutin, isoquercitrin, isochlorogenic acid B, isochlorogenic acid A, astragalin, isochlorogenic acid C, rosmarinic acid, and trans-cinnamic acid. Thus, AVTE appears to be an effectively functional drink due to its rich functional components and anti-aging activities.

## 1. Introduction

Senescence mainly refers to the degradation due to age of the cell, tissue, and organ structure and function, a process gradually increasing the body’s vulnerability to death, and can be caused by many factors, including genetics, environment, and diet [1,2]. At present, the mechanism of aging is not clear. The most widely accepted theory is the free radical theory, which states that as the human body ages, there is an increase in oxygen free radicals, creating an imbalance in the free radicals production system of the body. Excessive free radicals can cause lipid peroxidation, damage biological macromolecules, and lead to cell damage such as nuclear swelling, changes in chromatin structure, decrease or loss of cell replication ability, and slowdown of metabolism; eventually such damage can lead to physical aging and dysfunction, such as inflammation [3], respiratory disease [4], and cardiovascular disease [5].

Dozens of different aging mouse models have been established in order to study the path and pathogenesis of human aging and to evaluate its therapeutic effects. The mouse aging model is mainly divided into three types: Spontaneous, induced (including d-galactose), and transgenic. The d-galactose-induced aging mouse is a widely used model because of its simplicity and performance. Its excessive accumulation in the body can generate more free radicals through a series of reactions and actions, causing oxidative damage and inflammation, which can lead to diseases similar to human natural aging symptoms. Oxidative damage and inflammation accompany the entire aging process, and we can improve aging symptoms through antioxidant and anti-inflammatory treatments [6,7].

Common markers of oxidative aging levels in the body are malondialdehyde (MDA), superoxide dismutase (SOD), glutathione (GSH), glutathione peroxidase (GSH-Px), catalase (CAT), total antioxidant capacity (T-AOC), and nitric oxide (NO) [8]. Among these markers, aging is closely related to the body SOD activity and MDA content. MDA is the main product of lipid peroxidation and reflects the extent of oxidative damage, while SOD is widely found in living organisms and is a key enzyme in the body’s antioxidant system [9,10]. In addition to the body’s own resistance systems (such as antioxidant enzymes and proteins), antioxidants can also capture and stabilize free radicals such as ascorbic acid, vitamin E, polyphenols, and other exogenous small molecules to protect the body from free radical damage [11]. The cytokines IL-6, IL-12, TNF-α, and IL-1β play key roles in regulating immune responses, while aging is characterized by chronic low-grade inflammation. Therefore, the anti-aging effect can be evaluated by measuring inflammation levels [12].

*Apocynum venetum* tea is native to the Bayu region of southern China and has received much attention as a medicinal drink. Based on the degree of fermentation, the tea can be divided into three groups, each with very different biologically active components: Non-fermented tea (such as green tea), semi-fermented tea (such as oolong tea), and whole fermented tea (such as black tea) [13]. Tea polyphenols are the main active components in tea, and have many physiological functions such as anti-aging, antibacterial, and anti-tumor [14]. *Apocynum venetum* tea belongs to a non-Camellia tea family, its original leaves belonging to the Oleander family, and consists of three species: *Apocynum venetum* L, *Poacynum hendersonii* (Hook.f) Woodson, and *Poacynum pictum* (Sckrenk) Baill [15]. For this study, young leaves of *Apocynum venetum* L. were dried at an appropriate temperature and humidity, resulting in a unique flavor and taste as a result of the processing technique and a series of complex chemical changes. The study found that its leaves contained flavonoids, organic acids, amino acids, minerals, and other chemical components, which are involved in liver protection [16] and cancer prevention [17], as well as having anti-aging and antibacterial properties [15]. In this context, the aging model of the mice induced by d-galactose was used to evaluate the protective effect of AVTE and determine its bioactive compounds.

## 2. Materials and Methods

### 2.1. Preparation of AVTE

A sample of *Apocynum venetum* tea (Brand: Niamese; Company: Xinjiang Lvkang *Apocynum* Co., Ltd., Urumqi, Xinjiang, China), which originated in the Luobu Plain, Xinjiang, China, was pulverized, freeze-dried (−50 °C, 2.0 Pa), and subsequently ground into separate fine powders. A 10-fold volume of 70% ethanol solution was added to the powder sample, extracted in a water bath (60 °C for 1 h) twice, and the filtrates were merged. The extract was filtered with FL-3 macroporous resin, eluted with 70% ethanol until colorless, and evaporated using a rotary evaporator (N-1100; Eywla; Tokyo, Japan) to remove water and ethanol; the resulting AVTE was then freeze-dried (−50 °C, 2.0 Pa) and stored at 4 °C until needed.

### 2.2. Animals

Forty mice from Kunming (6 weeks old, male) were purchased from the Experimental Animal Center of Chongqing Medical University (Chongqing, China). The male mice were selected because there was no physiological cycle, and the hormones and enzyme activities in the body remained basically in dynamic balance, and the effect on the experimental results was smaller than that in the female mice. The mice were housed in a controlled facility at a constant temperature (25 ± 2 °C) and relative humidity (50 ± 5%) with a 12/12 h light/dark cycle and free access to a standard mice chow diet and water.

### 2.3. Induction of Aging Model in Mice

After the mice adapted to the environment for a week, they were divided into 4 groups (normal group, model group, low dose group, and high dose group), 10 in each group, in order to study the preventative effect of AVTE against the d-galactose-induced aging model. All groups were fed a normal diet and water for 6 weeks. The normal group received no additional treatment. The mice in the other 3 groups received 120 mg/(kg·d) of d-galactose (Sinopharm Chemical Reagent Co., Ltd., Shanghai, China) injected intraperitoneally from weeks 2 through 6. The mice in the low dose group received 250 mg/(kg·d) ATVE administered orally while the mice in the high dose group received 500 mg/(kg·d) ATVE, also administered orally. The volume of injection or oral administration was 0.2 mL once a day. This AVTE dose range was selected based on previous literature (LD_50_ >10.0 g/kg) and pre- experiment, which did not cause any toxic effects in the mice [18]. The protocol for these animal experiments was approved by the Animal Ethics Committee of Chongqing Collaborative Innovation Center for Functional Food, Chongqing University of Education (ethical approval code: 201903001B), Chongqing, China. 

### 2.4. Collection of Samples

At the end of 6 weeks, the mice fasted for 18 h after the last gavage and injection of d-galactose and were then killed. After blood collection, the liver, kidney, spleen, and heart were quickly separated, the surrounding fat and connective tissue were removed, the residual blood was washed, and the organs were accurately weighed. Part of the liver was fixed in a 10% formalin solution for making sections. The remaining parts and tissues were stored at −80 °C after freezing in liquid nitrogen for a follow-up experiment.

### 2.5. Histological Observations

The liver was immediately removed after sacrificing and fixed in 10% (*v*/*v*) buffered formaldehyde, paraffin embedded, and sectioned, before undergoing hematoxylin and eosin staining and observation with a BX43 microscope (Olympus, Tokyo, Japan).

### 2.6. Measurement of Biochemical Indicators in Serum

The blood from the mice was placed at 4 °C for 2 h then centrifuged for 15 min by freezing the centrifuge (3000 r/min, 4 °C). The upper serum was isolated and placed at −80 °C for future use. The malondialdehyde (MDA), nitric oxide (NO), superoxide dismutase (SOD), glutathione (GSH), glutathione peroxidase (GSH-Px), catalase (CAT), and total antioxidant capacity (T-AOC) in the upper serum were determined by assay kits (Nanjing Jiancheng Institute of Bioengineering, Jiangsu, China).

### 2.7. Measurement of Cytokine in the Serum

The levels of cytokines IL-6, IL-12, TNF-α, and IL-1β were determined by using enzyme-linked immunosorbent assay kits (ABCAM, Cambridge, MA, USA).

### 2.8. RT-qPCR Analysis

According to the manufacturer’s recommendation, total RNA from the liver tissue was isolated by Trizol reagent (Invitrogen, Carlsbad, CA, USA). The concentration and purity of RNA were determined at 260 nm by micro-ultraviolet-visible spectrophotometer, and the total RNA concentration of each group was adjusted to the same level. One microliter of oligo(dt)18 primer (500 ng) and 1.0 μL of total RNA (1.0 μg) were added to 10.0 μL of nuclease-free water and heated on a gradient PCR instrument for 5 min at 65 °C according to the manufacturer’s recommendations (RevertAid First-Strand cDNA Synthesis Kit; Thermo Fisher Scientific, Inc., Waltham, MA, USA). A mixed reagent containing 4.0 μL of 5× Reaction Buffer, 1.0 μL of Ribolock RNase Inhibitor (20 U), 2.0 μL of 10 mM dNTP Mix, and 1.0 μL of RevertAid Reverse Transcriptase (200 U/μL) was added to the total RNA system. The RNA was reverse transcribed into cDNA under the condition of 60 min at 42 °C and 5 min at 70 °C. The total reaction system (20 μL) consisted of 1.0 μL cDNA, 1.0 μL each of forward and reverse primers (10 μM), 10.0 μL premix (SYBR^®^ Select Master Mix; Thermo Fisher Scientific, Inc., Waltham, MA, USA), and 7.0 μL sterilized double-steamed water; it was mixed and reacted on an automatic thermal cycler (SteponePlus, Thermo Fisher Scientific, Inc., Waltham, MA, USA). The amplification conditions were as follows: Denatured at 95 °C for 3 min, annealed at 60 °C for 30 s, and extended at 95 °C for 1 min and cycled 40 times. The expression levels of the antioxidant genes SOD1, SOD2, CAT, and GSH1 were determined by RT-qPCR. The sequences of the related primers are shown in Table 1. The cDNA samples of each gene were amplified 3 times in parallel, and the mean value of Ct was used for analysis. The internal reference gene is GAPDH, and the levels of the relevant genes were calculated according to 2^−∆∆CT^ [19].

### 2.9. Chemical Standards

Isoquercitrin and astragalin were obtained from Sigma-Aldrich Chemical Co. (St. Louis, MO, USA). Chlorogenic acid, rosmarinic acid, and trans-cinnamic acid were obtained from Fluka Chemical Co. (Buchs, Switzerland). Neochlorogenic acid, cryptochlorogenic acid, isochlorogenic acid A, isochlorogenic acid B, and isochlorogenic acid C were purchased from Solarbio Technology Co. (Beijing, China). Rutin was obtained from Keli Technology Development Co. Guangzhou Testing Center (Guangzhou, China).

### 2.10. HPLC Analysis

The AVTE was dissolved in dimethyl sulfoxide (DMSO, for HPLC, Beijing Solarbio Science & Technology Co., Ltd., Beijing, China) to obtain a solution with a concentration of 10 mg/mL and diluted with 50% methanol to produce a final concentration of 2.5 mg/mL. The sample was passed through a 0.22-μm organic filter before testing. About 5 μL of the diluted AVTE sample solution was analyzed using an UltiMate3000 HPLC System (Thermo Fisher Scientific, Inc., Waltham, MA, USA) and separated using an Accucore C18 column (2.6 μm, 4.6 × 150 mm; Thermo Fisher Scientific, Inc., USA). Mobile phase A was water containing 0.5% acetic acid, and mobile phase B was acetonitrile. The flow rate was 0.5 mL/min with a column temperature of 30 °C, and the detection wavelength was 328 nm. The gradient elution conditions were as follows: Equilibrium stage was set for 10 min with 12% B (isocratic) then, 0–30 min with 12%–45% B (linear gradient), 30–35 min with 45%–100% B (linear gradient), and 35–40 min with 100% B (isocratic).

### 2.11. Statistical Analysis

The SPSS version 20.0 statistical software (SPSS Inc., Chicago, Illinois, USA) was used to analyze the experimental data. The results were analyzed using one-way analysis of variance (ANOVA) with Duncan’s multiple range tests, and *p* < 0.05 was considered statistically significant. All experiments were repeated 3 times, and the data were presented as the mean ± standard deviation (SD).

## 3. Results

### 3.1. Organ Coefficient

The organ coefficient can directly reflect the structural changes of the organ and indirectly reflect changes in organ function. Therefore, observing the changes of the organ coefficient in the mice had important reference value for judging mouse aging [20]. Compared with the normal group (Table 2), the coefficients of cardiac, liver, splenic, and kidney tissue in the aging model group decreased to a certain extent (*p* < 0.05). After treatment with different doses of AVTE, the coefficient of cardiac, liver, splenic, and kidney tissue were reduced to a certain extent, the degeneration of the organs in the mice was delayed, and the atrophy of the organs was antagonized to a certain extent [21].

### 3.2. Histological Analyses

**Figure 1:**
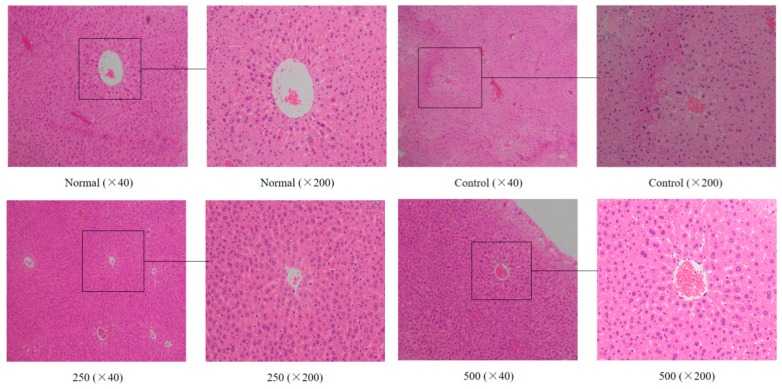
Effect of AVTE on the hepatic tissue morphology in the mice injured by d-galactose. Normal: Normal group; Control: Model control group induced by d-galactose, 120 mg/(kg·d); 250: Low dose group, 250 mg/kg; 500: High dose group, 500 mg/kg.

The liver plays an important role in the detoxification of potentially harmful chemicals and the regulation of a stable internal environment. It was found that the morphology of the liver tissue also changed during aging in the mice [22]. As shown in Figure 1, the liver cells of the normal group were regular in morphology and uniform in size and staining, while the hepatocyte cords were arranged in an orderly manner with clear boundaries and distributed radially around the central vein. In the aging group, the liver cells were not arranged regularly, the staining was uneven, the central venous traits were irregular, the hepatic cell lines were disordered, and the boundaries were unclear. Additionally, the cells were swollen, the cytoplasm was loose, and the cells showed some balloon-like changes, inflammatory cell infiltration, and some cell necrosis. After treatment with AVTE (250 and 500 mg/kg), the liver cells of the mice were again arranged in an orderly manner, with slight changes in morphology, while the hepatocyte edema, necrosis, and inflammation were all reduced [16].

### 3.3. MDA, SOD, GSH, GSH-Px, CAT, T-AOC, and NO Concentrations in Serum

**Figure 2:**
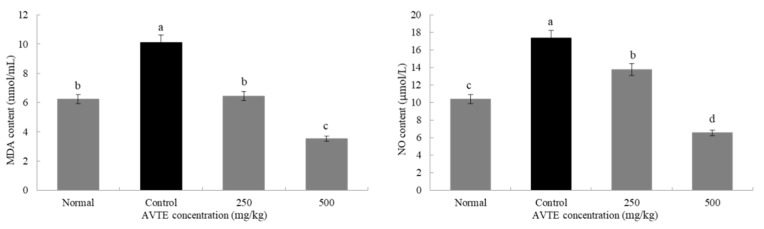
Effects of AVTE on malondialdehyde (MDA) and nitric oxide (NO) in serum of the mice. ^a–d^ Mean values with different letters in the same bar graph are significantly different (*p* < 0.05) according to Duncan’s multiple range test.

The activity of the antioxidant enzymes in the body decreases as the body ages while the accumulation of oxygen increases from base levels, eventually leading to abnormal cell structure and functional degeneration, resulting in a series of diseases [23]. As shown in Figure 2, compared with the normal group, the MDA and NO content in the serum of the model group mice were significantly increased with a difference that was statistically significant (*p* < 0.05). Compared with the model group, the MDA and NO content in the serum of the mice treated with the AVTE intervention groups (250 and 500 mg/kg) showed a statistically significant (*p* < 0.05) decrease.

**Figure 3:**
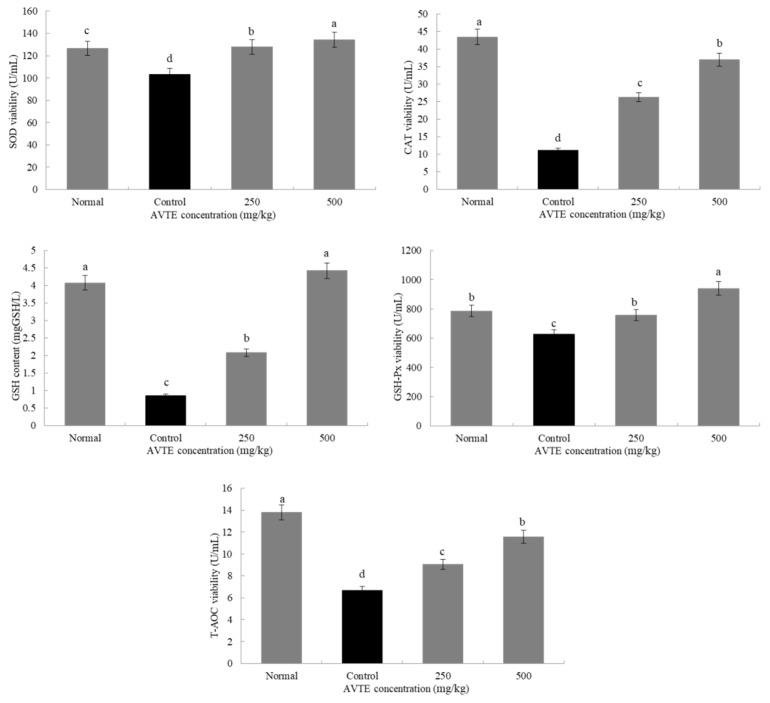
Effects of AVTE on superoxide dismutase (SOD), glutathione (GSH), GSH-peroxidase (Px), catalase (CAT) and total antioxidant capacity (T-AOC) in serum of the mice. ^a–d^ Mean values with different letters in the same bar graph are significantly different (*p* < 0.05) according to Duncan’s multiple range test.

Antioxidant enzymes (such as SOD, CAT, GSH-Px) can convert peroxides into low toxic substances or non-toxic water through redox action [8]. As shown in Figure 3, compared with the normal group, the SOD, GSH, GSH-Px, CAT, and T-AOC in the serum of the model group mice were significantly decreased (*p* < 0.05). Compared with the model group, after treatment with AVTE (250 and 500 mg/kg), the levels of antioxidants in the serum of the mice were significantly improved, and SOD GSH, GSH-Px, CAT, and T-AOC were significantly (*p* < 0.05) increased.

### 3.4. IL-6, IL-10, TNF-α and IL-1β Concentrations in Serum

**Figure 4:**
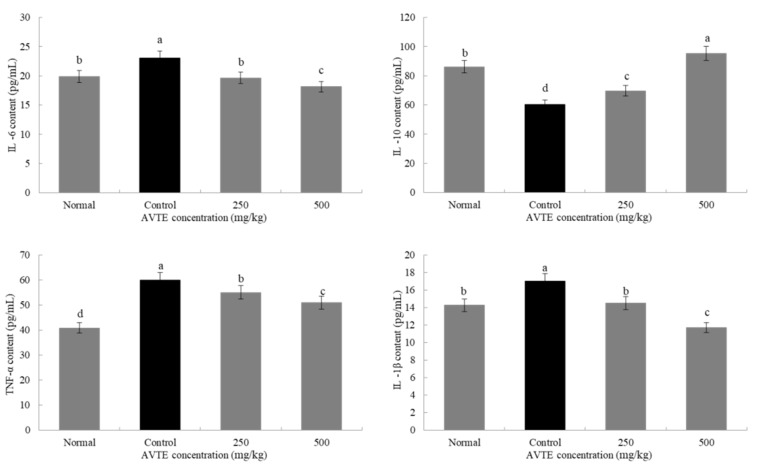
Effects of AVTE on tumor necrosis factor (TNF-α), interleukin (IL-6, IL-10, and IL-1β) in serum of the mice. ^a–d^ Mean values with different letters in the same bar graph are significantly different (*p* < 0.05) according to Duncan’s multiple range test.

Reactive oxygen species in the mice were activated in large amounts, which induced the expression of various cytokines such as tumor necrosis factor (TNF-α) and interleukins (IL-6, IL-10, and IL-1β) [24]. As shown in Figure 4, compared with the normal group, the expression of pro-inflammatory factors TNF-α, IL-6, and IL-1β in the serum of model group (d-galactose induced) increased (*p* < 0.05), especially the decrease of TNF-α, it can promote the production of various inflammatory factors by T cells, which is the key link of various signaling pathways [12]. In contrast, the expression of anti-inflammatory factor IL-10 decreased (*p* < 0.05). Compared with model group, low dose AVTE group (250 mg/kg) and high dose AVTE group (500 mg/kg) all had down-regulation effect on TNF-α, IL-6, and IL-1β, but up-regulation effect on IL-10, and the effect of the high dose AVTE group was more significant (*p* < 0.05), indicating that AVTE could reduce the expression of inflammation in the mice.

### 3.5. Effects of AVTE on the Gene Expression of SOD1, SOD2, GSH-Px, and CAT in the Liver of Aging Mice (RT-qPCR Assay)

**Figure 5:**
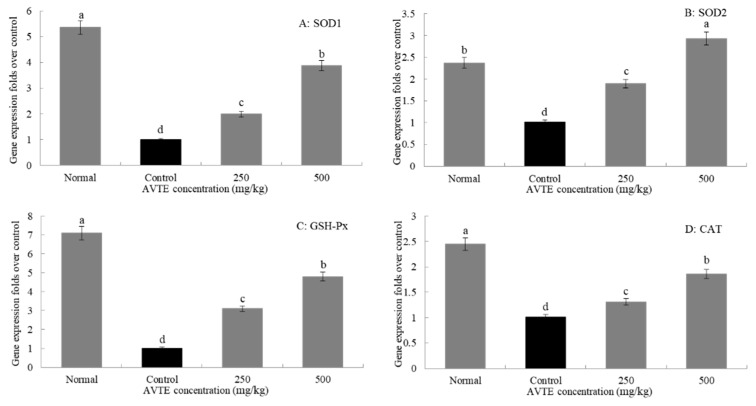
Effects of AVTE on the mRNA expression of SOD1, SOD2, GSH-Px, and CAT in liver tissue of the mice. ^a–d^ Mean values with different letters in the same bar graph are significantly different (*p* < 0.05) according to Duncan’s multiple range test.

As shown in Figure 5, the expression levels of SOD1, SOD2, GSH-Px, and CAT in the d-galactose-induced aging model group were significantly lower than those in the normal group. After treatment with different concentrations (250, 500 mg/kg) of AVTE, the expression of SOD1, SOD2, GSH-Px, and CAT in liver tissue was significantly improved, and the high dose of AVTE had the best effect (*p* < 0.05).

### 3.6. Analysis of the Chemical Composition of AVTE

**Figure 6:**
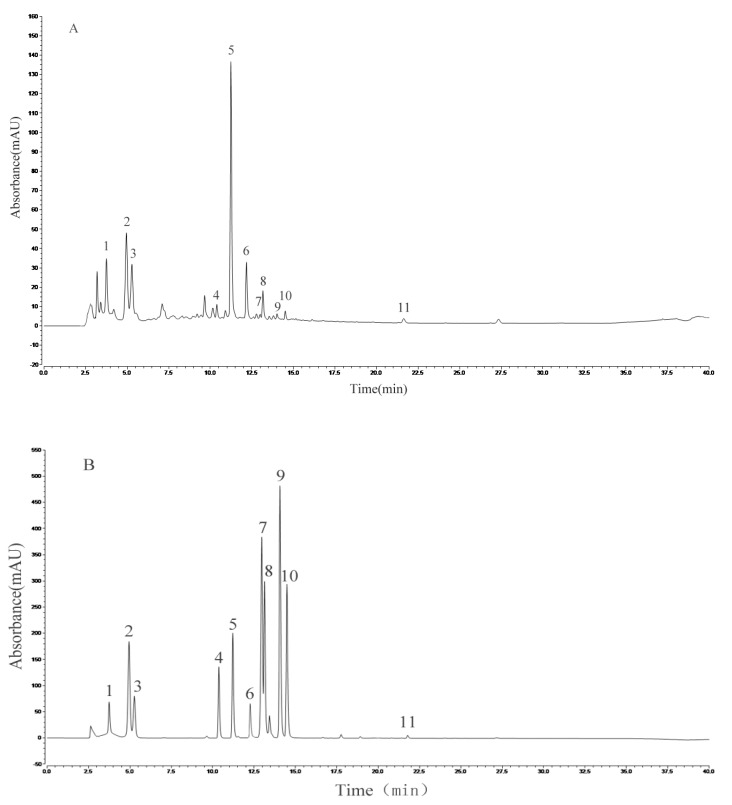
Analysis of the constituents of AVTE via high performance liquid chromatography (HPLC) assay. (**A**) AVTE chromatogram; (**B**) standard chromatograms.

**Figure 7:**
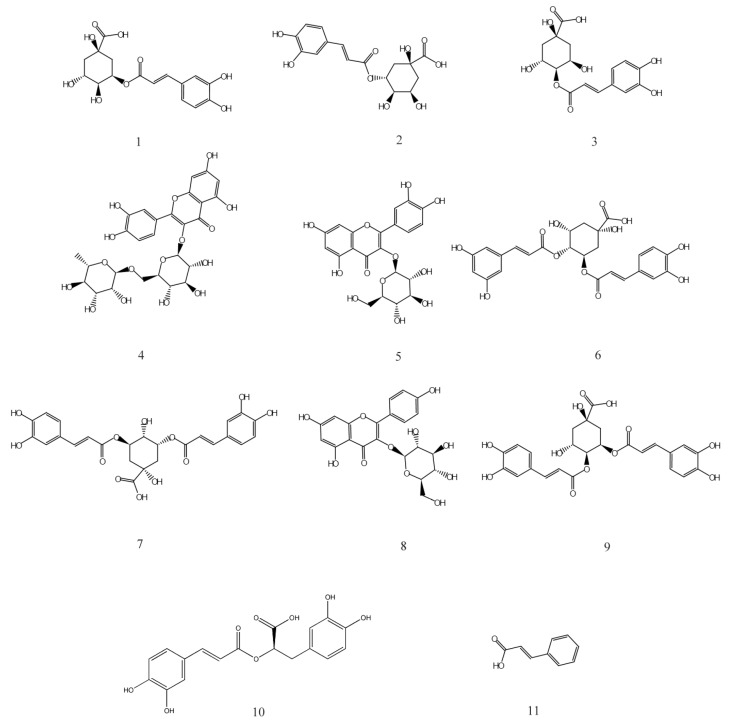
Chemical structures of 1–11 isolated from AVTE. 1: Neochlorogenic acid; 2: Chlorogenic acid; 3: Cryptochlorogenic acid; 4: Rutin; 5: Isoquercitrin; 6: Isochlorogenic acid B; 7: Isochlorogenic acid A; 8: Astragalin; 9: Isochlorogenic acid C; 10: Rosmarinic acid; 11: Trans-cinnamic acid.

HPLC analysis of the AVTE is shown in Figure 6A. Compared with the retention time of the chemical standards (Figure 6B), 11 peaks were identified from the AVTE, including neochlorogenic acid (peak 1, 3.743 min), chlorogenic acid (peak 2, 4.947 min), cryptochlorogenic acid, (peak 3, 5.270 min), rutin (peak 4, 10.273 min), isoquercitrin (peak 5, 11.213 min), isochlorogenic acid B (peak 6, 12.240 min), isochlorogenic acid A (peak 7, 12.963 min), astragalin (peak 8, 13.133 min), isochlorogenic acid C (peak 9, 14.063 min), rosmarinic acid (peak 10, 14.483 min), and trans-cinnamic acid (peak 11, 21.780 min). Figure 7 shows the structures of these chemicals.

## 4. Discussion

Many kinds of plants have been used as tea drinks in China since ancient times. Traditionally, “tea” refers to the buds, tender leaves, or leaves of the plants from the *Camellia* genus of the Theaceae family, while non-*Camellia* tea consists of different parts of the plants from different families and genera [25]. According to the degree of fermentation, the biologically active ingredients of tea vary greatly, but the main active ingredient in tea is polyphenol, a chemical known to act as a preventive agent against malignant tumors and have strong free radical scavenging and reducing activity. Non-fermented tea (e.g., green tea) has a higher polyphenol content, causing astringency and irritation to the stomach. Total fermented tea (e.g., black tea) has a greater loss of polyphenols, but its components are more complex (e.g., aromatic components), causing a better effect on stomach maintenance [26]. Studies have shown that *Apocynum venetum*, a non-*Camellia* tea, contains phenolic acids, flavonoids, amino acids, and other chemicals that have been shown to lower blood pressure, blood lipids, and blood sugar levels [15], have antioxidant and anti-inflammatory properties [16], and anti-cancer properties [17].

In this study, eleven bioactive components including chlorogenic acid and its five isomers were detected in AVTE by HPLC. Chlorogenic acids (CGAs) are phenolic acids with vicinal hydroxyl groups on aromatic residues, derived from the esterification of trans-cinnamic acids (including caffeic, ferulic and *p*-coumaric acids) with quinic acid. There are several CGA subgroups, including neochlorogenic acid, cryptochlorogenic acid, isochlorogenic acid A, isochlorogenic acid B, and isochlorogenic acid C. These compounds have many pharmacological actions, such as scavenging free radicals, lowering blood pressure and blood lipids, and protecting the liver and gallbladder. CGAs are abundant in the human diet, and epidemiological studies have shown that consumption of tea, coffee, wine, different herbal preparations, and some fruits (such as apples, pears, and certain berries) can reduce the risk of various chronic diseases [27,28].

Rosemarinic acid is a polyphenolic hydroxyl compound formed by the condensation of caffeic acid and 3,4-dihydroxyphenyl lactic acid. The biosynthesis pathway includes two parallel branching pathways: Phenylalanine and tyrosine. Its antioxidant activity is stronger than caffeic acid and chlorogenic acid. It helps to prevent cell damage caused by free radicals, reducing the risk of cancer and atherosclerosis. It also demonstrates anti-melanin production and anti-inflammatory, anti-mutagenic, and anti-peeling activity [29]. Trans-cinnamic acid is mainly used to treat coronary atherosclerosis and other diseases. It can inhibit the formation of black tyrosinase and significantly inhibit the proliferation of lung adenocarcinoma cells [30]. Rutin is a hydrogen transporter, which may participate in the role of oxidoreductase in vivo, affect thyroid activity, protect adrenaline from oxidation, enhance and promote vitamin C accumulation, maintain vascular elasticity, reduce vascular permeability and fragility, promote cell proliferation, and prevent blood cell agglutination. It has also been shown to have hypolipidemic and anti-inflammatory effects [31].

Isoquercitrin, a flavonol compound obtained by hydrolyzing rutin, widely exists in mulberry leaves, *Apocynum venetum*, *Cyclocarya paliurus*, *Thalictrum angustifolia*, and other medicinal plants. As an important active ingredient of *Apocynum venetum*, it has been shown to be anti-inflammatory [16] in addition to possessing anti-cancer [17] and anti-oxidation effects [32]. Astragalin is also a natural flavonoid widely existing in medicinal plants. It has anti-inflammatory and anti-hepatotoxic effects [33]. In conclusion, the presence of these bioactive chemicals in AVTE may be the main reason for their various pharmacological effects.

The long-term injection of d-galactose prevents the mice from completely metabolizing. When the excess d-galactose accumulates in the body, it can be reduced to galactitol, which has toxic effects on the body. The galactitol can then be further oxidized to galactose aldehyde and hydrogen peroxide by the action of its enzyme. Hydrogen peroxide, as a reactive oxygen species (ROS), can produce hydroxyl radicals after reaction, resulting in the decrease of antioxidant activity in vivo. The accumulation of free radicals in vivo is further aggravated by reactive oxygen species. d-galactose can also induce up-regulation of the intracellular Ca^2+^/Mg^2+^ ratio, mitochondrial dysfunction, and phospholipase A2 activation. In conclusion, d-galactose induces oxidative stress, induces the expression of many inflammatory cytokines and irreversible cell apoptosis, leading to aging and functional deterioration [34]. In most publications, the mice or rat aging model was established by intraperitoneal injection of 50–500 mg/(kg·d) d-galactose daily for 6–8 weeks [35]. We established an aging model of mice through intraperitoneal injection of 120 mg/(kg·d) d-galactose for six weeks in this research, and the effect was significant (sore whiskers). Therefore, we recommend low-dose, long-term modeling, which is more similar to natural aging. As the most vigorous organ of the human body, the liver is also the most important detoxification organ and is very sensitive to drug metabolism. Studies have shown that aging cells cause aging and age-related diseases by producing a low-grade inflammatory state [36,37,38]. d-galactose-induced aging in mice can cause hepatic cell swelling, necrosis, inflammatory cell infiltration, and other pathological changes, leading to liver damage, mainly oxidative stress damage and inflammatory response [39].

Under normal circumstances, the body’s oxidation and anti-oxidation systems are in dynamic equilibrium, and the body’s own antioxidant enzymes such as SOD, CAT, and GSH-Px play an important role in scavenging free radicals. When external stimuli cause oxidative stress damage, the body’s metabolism is disrupted, and the initial equilibrium state cannot be maintained; lipid peroxidation causes the body to produce a large amount of peroxidation products [5]. We found that the activities of antioxidant enzymes SOD, GSH-Px, and CAT in aged liver tissue were significantly lower than normal. On the contrary, the content of MDA and NO in peroxidation products was significantly higher than those in the normal group and the antioxidant system in the mice was unbalanced. MDA is the final metabolite of membrane lipid peroxidation in vivo, which can better reflect the degree of tissue peroxidation. The MDA released on the cell membrane can react with proteins and nucleic acids to cause cross-linking polymerization and also inhibit the synthesis of proteins, mainly by damaging the membrane structure and function and changing its permeability, thereby affecting the biochemical reaction of normal organisms [40]. NO is a highly reactive free radical in the body that relaxes vascular smooth muscle, inhibits platelet aggregation, and mediates cytotoxic effects and immune regulation. Its abnormality is closely related to the development of certain diseases, and its concentration increases with the age of the organism [41].

SOD is an important enzyme in the antioxidant system of the body. According to the combination of different metal ions, SOD is divided into Mn-SOD, Fe-SOD, and Cu/Zn-SOD. It can convert the excess superoxide anion radical of the body into hydrogen peroxide, which is converted into H_2_O by CAT and GSH-Px to protect it from damage. With the aging of the body, the activity of SOD is continuously decreasing [10]. Compared with the aging group, the levels of SOD in the AVTE group (250 mg/kg and 500 mg/kg) increased, and the high dose group were significantly better, indicating that AVTE can alleviate lipid peroxidation in mice and alleviate the degree of aging damage in mice. CAT can regulate superoxide anion radicals in the body and has a high affinity for hydrogen peroxide. It can reduce toxic hydrogen peroxide to H_2_O, and coordinate with SOD to delay the aging of the body [42]. Under the action of GSH-Px, GSH can reduce intracellular hydrogen peroxide to form H_2_O, and GSH is oxidized to GSSG, which generates GSH under the catalysis of glutathione reductase. GSH-Px works together with GSH to protect the body from ROS damage and maintain the normal function of the body [43]. T-AOC can scavenge reactive oxygen species to put the body in a redox state, making it a good indicator of the comprehensively reflecting enzymes and non-enzymatic antioxidants [44]. The interaction of multiple compounds in AVTE may be the main reason for its ability to significantly regulate changes in these indicators in aged mice.

d-galactose-induced mouse aging not only causes oxidative stress damage in the body, resulting in low immune function, but also secretes inflammatory cytokines such as TNF-α and IL, causing apoptosis and necrosis of hepatocytes [45]. The immune system is associated with Th1 or Th2 cells and contains both anti-inflammatory and pro-inflammatory cytokines, which play an important role in the inflammatory response. Orchestrating cell-mediated immunity is a vital function of Th1 cells, which secrete INF-γ, IL-2, and IL-12. In contrast, regulation of humoral responses is a key capability of Th2 cells, which secrete IL-4, IL-6, and IL-10. These subpopulations are regulated by important cytokines [46]. INF-γ can suppress the development of Th2, while IL-4 and IL-10 cells inhibit the Th1 response; their mutual regulation creates a normal state. This homeostasis is disrupted during inflammation. Therefore, we screened pro-inflammatory cytokines IL-1β, TNF-α, and IL-6, which cause immune disorders and amplify inflammation, as well as anti-inflammatory cytokine IL-10. IL-1β is an important pro-inflammatory factor, which can induce multiple signaling pathways in cells and promote the production of cytokines. It is considered to be one of the most potent inflammatory factors [47]. IL-6 can promote the accumulation of acute proteins and T cells in the inflammation site, and TNF-α is the earliest and most important mediator in the inflammatory process—they both play an important role in the pathological process of liver injury. They can act on hepatocyte surface related receptors, have obvious toxicity to the liver, and cause massive necrosis of liver cells; inhibition of TNF-α and IL-6 expression can attenuate liver aging damage caused by d-galactose [48,49,50]. In this study, AVTE can attenuate the expression of inflammatory factors in aging mice by down-regulating TNF-α, IL-6 and IL-1β, and up-regulating IL-10.

Cardio-vascular functions changes are also major symptoms of aging. The most significant effect was manifested in left ventricular hypertrophy and left atrial dilatation. Fibrosis occurred with long-term myocardial insufficiency, arrhythmia, and myocardial degenerative lesions occurred, and the final outcome was heart failure [51]. It can also cause damage to the intima of the aortic valve, causing blood eddy, which in turn leads to endocarditis. This leads to mitral annular calcification (MAC), which is closely related to conduction system disease, atherosclerosis, and adverse cardiovascular disease [52]. The roots of cannabis *Apocynum cannabium* and *Apocynum androsaemifolium* in the first half of the 20th century were used to treat heart disease in Europe [53]. In recent years, an in vitro study showed that *Apocynum venetum* extract increased the contractile force and pulses of isolated guinea pig atria [54]. Although the cardiovascular function in this study has not been directly tested and proved, the changes of MDA, SOD, GSH, GSH-Px, CAT, T-AOC, and NO in the serum of the mice were analyzed, which are related to cardiovascular function. Further, those compounds identified by HPLC from AVTE have been shown to improve cardiovascular function.

In addition, we clarify the limitations and precautions of this research, and give the direction for future research. The AVTE treatment group was designed into the mice grouping scheme and could serve the entire experiment better. Although the solubility of DMSO to AVTE is stronger than water, ethanol, etc., some insoluble compounds with DMSO are still neglected when detected by HPLC. Mass spectrometry analysis is more convincing than single HPLC analysis for the identification of compounds. The biological activity and functional expression of AVTE at the protein level are still unclear. However, the research on *Apocynum venetum* tea (non-Camellia tea) is relatively rare compared with the typical tea, especially as the functional evaluation in vivo of AVTE is less. We evaluated the effect of AVTE on d-galactose-induced aging mice, and the improvement was significant. The compounds were analyzed, and some meaningful compounds were found, which were not found in other studies. We will further improve them in future experiments, and we plan to study the effects and specific mechanisms of AVTE on cardiovascular function in aging mice, and to evaluate the pharmacodynamics of multiple compounds combination in AVTE.

## 5. Conclusions

In summary, anti-aging effects of AVTE (250, 500 mg/kg) include the regulation of the weight of the major organs; improvement of hepatocyte morphology, edema, and inflammation; up-regulation of SOD, CAT, GSH, GSH-Px, T-AOC; down-regulation of NO and MDA levels; reductions in the concentration of pro-inflammatory factors (IL-6, TNF-α and IL-1β), and increases in the concentration of anti-inflammatory factor IL-10. In addition, the 11 compounds tested by HPLC were previously shown to have many biological activities including anti-oxidant, anti-inflammatory, and anti-cancer, etc.

## Figures and Tables

**Table 1:** The sequences of reverse transcription-polymerase chain reaction primers.

Gene Name	Sequence
SOD1	Forward: 5′-AACCAGTTGTGTTGTGAGGAC-3′
	Reverse: 5′-CCACCATGTTTCTTAGAGTGAGG-3′
SOD2	Forward: 5′-CAGACCTGCCTTACGACTATGG-3′
	Reverse: 5′-CTCGGTGGCGTTGAGATTGTT-3′
CAT	Forward: 5′-GGAGGCGGGAACCCAATAG-3′
	Reverse: 5′-GTGTGCCATCTCGTCAGTGAA-3′
GSH-Px	Forward: 5′-GTCGGTGTATGCCTTCTCGG-3′
	Reverse: 5′-AGAGAGACGCGACATTCTCAAT-3′
GAPDH	Forward: 5′-AGGTCGGTGTGAACGGATTTG-3′
	Reverse: 5′-CTGCAGCTCGTTCATCTGGG-3′

**Table 2:** Effects of Apocynum venetum tea extract (AVTE) on organ coefficient in aging mice induced by d-galactose.

Organs	Normal (mg/g)	Control (mg/g)	250 (mg/g)	500 (mg/g)
Heart	6.41 ± 0.11 ^aA^	5.95 ± 0.06 ^b^	6.39 ± 0.08 ^a^	6.64 ± 0.12 ^a^
Liver	38.61 ± 3.22 ^a^	36.89 ± 1.3 ^b^	37.86 ± 2.08 ^a^	38.69 ± 2.69 ^a^
Spleen	2.58 ± 0.03 ^b^	2.1 ± 0.14 ^c^	2.62 ± 0.11 ^b^	2.94 ± 0.17 ^a^
Kidney	16.18 ± 1.64 ^a^	13.66 ± 1.56 ^b^	15.23 ± 1.23 ^a^	16.46 ± 2.18 ^a^

^a–c^ Mean values with different letters in the same line are significantly different (*p* < 0.05) according to Duncan’s multiple range test. ^A^ Values are mean ± SD of different organ coefficient. Organ coefficient (mg/g) = organ weight (mg)/body weight (g).
